# Editorial: Model organisms in toxicology

**DOI:** 10.3389/ftox.2025.1560492

**Published:** 2025-02-18

**Authors:** Michael Aschner, Asok K. Dasmahapatra, Arantza Muriana

**Affiliations:** ^1^ Pediatrics, New York, TN, United States; ^2^ Department of Biomolecular Sciences, Environmental Toxicology Division, University of Mississippi, Oxford, MS, United States; ^3^ Biobide, Donostia-San Sebastian, Spain

**Keywords:** model organisms, toxicology, genetics, endocrinology, risk assessment, fish

## Introduction

Model organisms are invaluable tools for understanding the mechanisms and consequences of toxic exposures. They provide ethically viable and scientifically robust alternatives to mammalian models, enabling researchers to study biological processes with relevance to human health while avoiding direct human exposure to harmful substances. From simpler organisms like *Caenorhabditis elegans* and *Drosophila melanogaster* to vertebrates such as zebrafish (*Danio rerio*) and medaka (*Oryzias latipes*), these models offer insights into toxicological processes, including gene-environment interactions, endocrine disruption, and chemical susceptibility (Wittbrodt et al., 2002; Novelli et al., 2012; Howe et al., 2013; Schartl, 2013; Bailik-Meisner et al., 2018; Jaka et al., 2023; Weiner et al., 2023).

In recent years, the use of some of those organisms has gained significant attention due to its alignment with the principles of the 3 Rs—Refinement, Replacement, and Reduction—of animal experimentation. For example, under EU Directive 2024/1262, zebrafish and medaka embryos are not considered animals in experimentation until they reach 5–6 days post-fertilization, making them a valuable alternative in the study of toxicology. Their early developmental phase, during which they are transparent, and their organs are visible, allows for refined monitoring of toxicity without the ethical concerns that arise from the use of higher vertebrates. Fish embryos, therefore, represents *in vivo* new alternative models (NAM) that reduces the need for traditional animal models, and their use aligns with current efforts to minimize the number of animals used in research, as non-invasive observation makes them a valuable tool in toxicological studies, environmental monitoring, and pharmacological research, while complying with EU regulations on animal welfare.

This Research Topic in *Frontiers in Toxicology*—“Model Organisms in Toxicology”—showcases four manuscripts that underscore the utility of fish models in toxicological studies. These articles explore the potential of zebrafish and medaka, among other species, in advancing our understanding of the effects of environmental and pharmaceutical toxicants.

The first manuscript, “Leveraging a high-throughput screening method to identify mechanisms of individual susceptibility differences in a genetically diverse zebrafish model,” by Wallis et al., investigates gene-environment interactions using zebrafish embryos exposed to abamectin, a neurotoxic insecticide. The findings highlight zebrafish’s adaptability as a model for identifying mechanisms underlying individual susceptibility to chemical exposure, offering critical insights into population-level variability in toxicological outcomes.

The second manuscript, “Ex blind: bridging the gap between drug exposure and sex-related gene expression in Danio rerio using next-generation sequencing (NGS) data and a literature review to find the missing links in pharmaceutical and environmental toxicology studies,” by King and Zenker, employs zebrafish as a model for studying sex-determining gene expression during early development. By identifying genes related to sex differentiation, this research has implications for sex-specific responses to drug exposure and paves the way for more targeted therapeutic strategies and hormonal effects on them.

The third article, “A systematic review of the evaluation of endocrine-disrupting chemicals in the Japanese medaka (Oryzias latipes) fish,” by Dasmahapatra et al., explores medaka as a complementary model to zebrafish for studying endocrine-disrupting chemicals (EDCs) in aquatic environments. This review provides evidence of medaka’s utility in identifying EDCs that impact human and environmental health, reinforcing its significance in toxicological research.

The fourth manuscript, “A systematic review of the toxic potential of parabens in fish,” by Dasmahapatra et al., examines mainstream (zebrafish, medaka) and non-mainstream fish species (common carp, fathead minnows, Nile tilapia, and rainbow trout) for their responses to paraben exposure. Parabens, widely used as preservatives in cosmetics and pharmaceuticals, are identified as endocrine disruptors targeting critical hormonal pathways. The review highlights their long-lasting environmental persistence, intergenerational toxicity, and behavioral effects on aquatic organisms, emphasizing the need for stricter regulatory oversight on EDCs, a perfect closure looking to the future of fish application in environmental toxicology especially in endocrine disruption.

## Significance of fish models in toxicology

Fish models, particularly zebrafish and medaka, have become indispensable alternative methodologies for toxicological studies due to their genetic similarities to humans, short life cycles, and suitability for high-throughput assays (Wittbrodt et al., 2002; Howe et al., 2013). Furthermore, they are aligned with ethical guidelines in animal research, thus enhancing their significance within toxicological studies. These models provide a controlled platform for studying chemical safety, toxicological mechanisms, and environmental risks. Recent advancements in genomics and bioinformatics have expanded the potential of non-mainstream fish species, enabling researchers to explore evolutionary adaptations and their implications for human health.

In addition to their role in environmental toxicology, fish models contribute to understanding complex phenomena such as gene-environment interactions, endocrine disruption, and sex-specific responses. These insights are crucial for developing targeted therapeutic interventions and informing regulatory frameworks aimed at protecting human and environmental health.

## Conclusion

The articles published in this Research Topic illustrate the critical role of fish models, particularly zebrafish and medaka, in toxicology. These studies shed light on the mechanisms underlying gene-environment interactions, sex determination, the impact of endocrine-disrupting chemicals, and the toxic potential of parabens in aquatic environments. Together, they highlight the versatility and relevance of fish models in addressing pressing toxicological questions, while complying with EU regulations on animal welfare.

As advancements in high-throughput screening, sequencing technologies, and computational modeling continue, the potential of fish and other non-traditional models will only expand. These organisms will remain at the forefront of toxicological research, advancing our understanding of chemical safety and contributing to strategies for mitigating risks to human and environmental health.

## References

[B1] Bailik-Meisner M. Truong L. Scholl E. H. La Du J. K. Tanguay R. L. (2018). Elucidating gene-by-environment interactions associated with differential susceptibility to chemical exposure. Environ. Health Persp 126. 10.1289/EHP2662 PMC6084885 29968567

[B2] Howe K. Clark M. D. Torroja C. F. Torrance J. Berthelot C. Muffato M. (2013). The zebrafish reference genome sequence and its relationship to the human genome. Nature 496 (7446), 498–503. 10.1038/nature12111 23594743 PMC3703927

[B3] Jaka O. Iturria I. Martí C. de Mendoza J. H. Mazón-Moya M. J. Rummel C. (2023). Screening for chemicals with thyroid hormone-disrupting effects using zebrafish embryo. Reprod. Toxicol. 121. 10.1016/j.reprotox.2023.108463 37619763

[B4] Novelli A. Vieira B. H. Cordeiro D. Cappelini L. T. Vieira E. M. Espindola E. L. (2012). Lethal effects of abamectin on the aquatic organisms, *Daphnia similis, Chironomus xanthus,* and *Denio rerio. Chemosphere* . Chemosphere 86, 36–40. 10.1016/j.chemosphere.2011.08.047 21955349

[B5] Schartl M. (2013). Beyond the zebrafish: diverse fish species for modeling human disease. Dis. Model Mech. 21, 181–192. 10.1242/dmm.012245 PMC3917239 24271780

[B6] Weiner A. M. J. Irijalba I. Gallego M. P. Ibarburu I. Sainz L. Goñi-de-Cerio F. (2023). Validation of a zebrafish developmental defects assay as a qualified alternative test for its regulatory use following the ICH S5(R3) guideline. Reprod. Toxicol. 123. 10.1016/j.reprotox.2023.108513 38016617

[B7] Wittbrodt J. Shima A. Schartl M. (2002). Medaka: a model organism from the far east. Nat. Rev. Genet. 3, 53–64. 10.1038/nrg704 11823791

